# Impact of ATM rs1801516 on late skin reactions of radiotherapy for breast cancer: Evidences from a cohort study and a trial sequential meta-analysis

**DOI:** 10.1371/journal.pone.0225685

**Published:** 2019-11-22

**Authors:** Salvatore Terrazzino, Sarah Cargnin, Letizia Deantonio, Carla Pisani, Laura Masini, Pier Luigi Canonico, Armando A. Genazzani, Marco Krengli

**Affiliations:** 1 Department of Pharmaceutical Sciences and Centro di Ricerca Interdipartimentale di Farmacogenetica e Farmacogenomica (CRIFF), University of Piemonte Orientale, Novara, Italy; 2 Radiation Oncology Clinic, Oncology Institute of Southern Switzerland, Bellinzona-Lugano, Bellinzona, Switzerland; 3 Radiotherapy, University Hospital Maggiore della Carità, Novara, Italy; 4 Department of Translational Medicine, University of Piemonte Orientale, Novara, Italy; Public Health England, UNITED KINGDOM

## Abstract

The relationship between the ataxia-telangiectasia mutated (ATM) rs1801516 gene polymorphism and risk of radiation-induced late skin side effects remains a highly debated issue. In the present study, we assessed the role of ATM rs1801516 as risk factor for radiation-induced fibrosis and telangiectasia, using the LENT-SOMA scoring scale in 285 breast cancer patients who received radiotherapy after breast conserving surgery. A systematic review with meta-analysis and trial sequential analysis (TSA) was then conducted to assess reliability of the accumulated evidence in breast cancer patients. In our cohort study, no association was found between ATM rs1801516 and grade ≥ 2 telangiectasia (GA+AA vs GG, HR_adjusted_: 0.699; 95%CI: 0.273–1.792, P = 0.459) or grade ≥ 2 fibrosis (GA+AA vs GG, HR_adjusted_: 1.175; 95%CI: 0.641–2.154, P = 0.604). Twelve independent cohorts of breast cancer patients were identified through the systematic review, of which 11 and 9 cohorts focused respectively on the association with radiation-induced fibrosis and radiation-induced telangiectasia. Pooled analyses of 10 (n = 2928 patients) and 12 (n = 2783) cohorts revealed, respectively, no association of ATM rs1801516 with radiation-induced telangiectasia (OR: 1.14; 95%CI: 0.88–1.48, P = 0.316) and a significant correlation with radiation-induced fibrosis (OR: 1.23; 95%CI: 1.00–1.51, P = 0.049), which however did not remain significant after TSA adjustment (TSA-adjusted 95%CI: 0.85–1.78). These results do not support an impact of ATM rs1801516 on late skin reactions of radiotherapy for breast cancer, nevertheless further large studies are still required for conclusive evidences.

## Introduction

Late skin reactions of radiotherapy for breast cancer mainly include subcutaneous fibrosis and telangiectasia, which may develop months to years following radiation treatment and have a negative impact on patients' quality of life [1, 2]. A considerable interindividual variability occurs in the development of late skin radiation injury, which depends not only on radiotherapy parameters such as total dose, dose per fraction, irradiated volume and dose inhomogeneity [3], but also on patient related factors such as age, life style and genetic background [4, 5]. Results of genome-wide association studies (GWASs) currently support the concept of normal tissue radiosensitivity as a complex trait resulting from the combined effects of multiple polymorphic gene variants, each with relatively modest effect [6, 7]. Thus, one single study with limited sample size has insufficient statistical power to detect gene polymorphisms with a small effect [8]. Nevertheless, a meta-analytic approach based on pooling data from multiple studies may be used to get adequate power for investigation of association between candidate gene polymorphisms and radiation-induced adverse effects on normal tissue [9–11].

The ataxia-telangiectasia mutated (ATM) protein, a serine/threonine protein kinase belonging to the phosphatidylinositol 3-kinase family, is known to be involved in the cellular response to DNA double-strand repair breaks [12, 13]. Mutations in the ATM gene are recognized to cause ataxia-telangiectasia, a complex genetic neurodegenerative disorder characterized by cerebellar degeneration, telangiectasia, immunodeficiency, cancer susceptibility and increased radiation sensitivity [14]. The observation that patients affected by ataxia-telangiectasia are extremely sensitive to ionizing radiation [15, 16] has provided the rationale to assess the role of ATM genetic variation on normal tissue radiosensitivity [17–19]. While there is a general consensus on the importance of ATM kinase protein in the individual radiation response, the contribution of ATM gene polymorphisms on clinical radiosensitivity is still controversial. The single nucleotide polymorphism (SNP) rs1801516 (also known as G5557A or D1853N) is the most commonly investigated variant of the ATM gene, which results in the non-conservative substitution of aspartic acid to asparagine at the amino acid position 1853 in exon 37. Three systematic reviews and meta-analyses have already addressed in cancer patients the impact of ATM rs1801516 on normal tissue injuries after radiation therapy, of which two showed a significantly increased risk of acute toxicity (total patients: n = 1588) [20] and radiation-induced fibrosis (total patients: n = 2000) [21] respectively among carriers of minor allele of rs1801516, while one other found no significant association with radiation-induced adverse effects in general [22]. On the other hand, trial sequential analysis (TSA) can be used to control both type I (false positive) and type II (false negative) errors of conventional meta-analyses [23], however no meta-analysis with TSA has been conducted so far to provide a reliable conclusion on the association between ATM rs1801516 and normal tissue toxicity after radiation treatment.

In order to clarify the role of ATM rs1801516 as genetic determinant for late side effects of radiotherapy for breast cancer, in the present study we firstly examined its association with radiation-induced fibrosis or telangiectasia in a cohort of breast cancer patients who received radiotherapy after breast conserving surgery. A systematic review and meta-analysis was then conducted to quantitatively estimate the effect of ATM rs1801516 on the risk of breast cancer patients to develop radiation-induced fibrosis or telangiectasia. We finally applied TSA to the meta-analytic results to establish whether the accumulated evidence on the association of ATM rs1801516 with late skin complications of radiotherapy for breast cancer might be sufficient to draw reliable conclusions.

## Material and methods

### Cohort study

This study comprised 285 Caucasian patients affected by histological confirmed breast cancer who underwent conservative surgery and adjuvant RT from 1989 to 2010 at our Department of Radiation Oncology. Radiotherapy technique was planned on computed tomography (CT) slices and consisted of two opposed tangential wedged beams to deliver a total dose of 50 Gy with daily fractionation of 2 Gy. An additional electronic boost dose on tumour bed was given in case of invasive lesions. At the time of patient recruitment, a peripheral blood sample was taken and stored at 4° C until genotyping. The onset of subcutaneous and cutaneous late toxicities, with special attention to telangiectasia and fibrosis, was assessed at annual follow-up visits. Late radiation-induced adverse effects were scored according to Late effects of Normal Tissue-Subjective Objective Management Analytical (LENT-SOMA) scale [24]. This study was approved by the local Ethics Committee (Comitato Etico Interaziendale–Azienda Ospedaliero-Universitaria “Maggiore della Carità”, Novara, Italy, CE 117/09) and conducted in accordance with the Declaration of Helsinki. All patient records data were fully anonymized as requested by the Ethics Committee. A written informed consent was obtained from all patients that agreed to have data from their medical records used in research, before study participation.

### Genotyping

Genomic DNA was extracted from 200 μl of whole blood with the QiaAmp DNA Mini Kit (Qiagen, Milan, Italy). ATM rs1801516 (5557G>A, D1853N) polymorphism was determined using TaqMan SNP genotyping assay (C__26487857_10) from Applied Biosystems (Milan, Italy). Real-time PCR amplification and detection of ATM rs1801516 was performed on a CFX Connect Real–Time PCR Detection System (Bio-Rad, Milan, Italy). Each real-time PCR amplification included no-template controls as well as positive controls for the 3 genotypes. About 20% of randomly chosen samples were re-genotyped for quality control and no discrepancies were found in the genotyping results.

### Statistical analysis

Deviation of ATM rs1801516 from the Hardy-Weinberg equilibrium (HWE) was assessed using the Pearson’s chi-square test as implemented in the Finetti’s program (http://ihg.gsf.de/cgi-bin/hw/hwa1.pl). The time to event end-point (grade ≥ 2 telangiectasia or grade ≥ 2 fibrosis) was calculated from the first session of RT; patients not experiencing the end-point of interest were censored at the last follow-up performed. The cumulative incidence of radiation-induced telangiectasia or fibrosis was calculated by the Kaplan–Meier method and comparisons between genotype groups were performed using the log-rank test. Cox regression analyses were performed to calculate the hazard ratio (HR) and the 95% confidence interval (CI) for the association of ATM rs1801516 with grade ≥ 2 telangiectasia or grade ≥ 2 fibrosis. Adjustments were also made for confounding clinical variables (P ≤ 0.05 in respective univariate analyses). Statistical analyses were performed using MedCalc software version 13.3.3 (MedCalc Software, Mariakerke, Belgium) and the significance threshold was set at P <0.05.

### Systematic review

We carried out computerized literature searches on PubMed, Web of Knowledge, Cochrane and Open Grey databases (last search up to August 26^th^, 2019) by using the Boolean combinations of the key terms: (breast neoplasm OR breast tumour OR breast tumor OR breast cancer OR breast carcinoma OR human mammary neoplasm OR human mammary tumour OR human mammary tumor OR human mammary carcinoma OR human mammary cancer) AND (radiotherapy OR radiation OR radiation therapy OR chemoradiotherapy) AND (polymorphism OR polymorphisms OR SNP OR SNPs OR variant OR variants OR genotype) AND (ATM OR Ataxia Telangiectasia Mutated) AND (fibrosis OR telangiectasia OR late reactions OR late toxicity). Eligible studies were required to meet the following inclusion criteria: (i) primary studies investigating the association of ATM rs1801516 or proxy SNPs (r^2^ = 1.0) with development of radiation-induced skin fibrosis or telangiectasia in breast cancer patients (ii) reporting sufficient data for estimating an odds ratio (OR) for the association with ATM rs1801516. Exclusion criteria were: narrative reviews, systematic reviews with or without meta-analysis, case reports and editorials; duplication of previous publications; not human studies. The potentially relevant articles were then read in their entirety to assess their appropriateness for inclusion in the meta-analysis. Reference lists of retrieved studies were also checked to identify other potentially eligible studies. The following data were extracted from each included study: the first author’s last name, year of publication, cohort name if any, study location, sample size, type of late adverse effect analysed, length of follow-up, detection method of ATM rs1801516, its minor allele frequency (MAF) and HWE p-value, event proportion in carriers and not carriers of rs1801516 A allele (GA or AA vs GG). If two or more studies shared part of the same patients’ population, the one with the larger sample size or more complete data was included. All studies were independently analyzed by two reviewers (S.T. and S.C.) and any discrepancies in data extraction were resolved through consensus. Methodological quality of the studies included in the systematic review was assessed independently by two authors (S.C. and S.T.) using the Newcastle–Ottawa scale (NOS) for cohort studies (available at: http://www.ohri.ca/programs/clinical-epidemiology/oxford-asp). The NOS assigns up to a maximum of 9 points, representing the highest methodologic quality. Studies with a NOS score > 7 were considered of high quality.

### Meta-analysis and trial sequential analysis

ORs were combined based on the dominant genetic contrast of ATM rs1801516 by using the random-effects (DerSimonian–Lairdmethod) model, which takes into account both within-study variance and cross-study variance [25]. In case of lack of heterogeneity, the random-effects model coincides with the fixed-effect model [26]. Heterogeneity between studies was tested using the Q statistic, with a p-value <0.10 indicating the presence of heterogeneity among studies. Heterogeneity was also quantified by the I^2^ metric, with I^2^ values >50% indicating high heterogeneity [27]. The presence of small-study effects and publication bias was evaluated graphically by funnel plots and statistically by means of the Egger’s test (P for significant asymmetry <0.1) [28]. Leave-one-out sensitive meta-analyses were performed to assess the robustness of pooled estimates by excluding individual cohorts one at a time and recalculating the pooled OR estimates for the remaining cohorts. Meta-regression analyses were also conducted to test the influence of late event incidence on the pooled overall estimate. All analyses were performed using ProMeta software (version 2; Internovi di Scarpellini, Daniele SAS, Cesena, Italy) and the significance of pooled estimates was set at P <0.05. Trial sequential analysis (TSA) was conducted to control type I and type II errors of conventional meta-analysis and to calculate the required information size (RIS) for a conclusive evidence [29]. TSA was performed using diversity-adjusted information size based on α = 0.05, β = 0.20 (power at 80%) through TSA software version 0.9.5.10 beta [30]. Diversity (D^2^) and event proportion among carriers and not carriers of rs1801516 A allele (GA or AA vs GG) were set based on the results of respective meta-analyses. TSA results were interpreted according to Brok et al. [29, 31].

## Results

### Cohort study

Detailed demographic, clinical and radiotherapy data in the whole cohort of breast cancer patients are shown in Table 1. Overall, 26 of the 285 breast cancer patients (9.1%) experienced moderate to severe telangiectasia (LENT-SOMA ≥ grade 2), while 51 subjects (17.9%) developed moderate to severe fibrosis (LENT-SOMA ≥ grade 2).

**Table 1:** Clinical and demographic characteristics of breast cancer patients (n = 285).

Variable	Mean (SD)	Number of patients (%)
**Age, years**	60.8 (10.1)	
**BMI, mean**	25.0 (3.8)	
**Breast diameter, cm**	12.2 (2.6)	
**Breast CTV, cc**	394.2 (530.0)	
**Diabetes mellitus**		
No		267 (93.7)
Yes		18 (6.3)
**Hypertension**		
No		211 (74.0)
Yes		74 (26.0)
**Vascular disease**		
No		263 (92.3)
Yes		22 (7.7)
**Tabagism**		
Never		242 (84.9)
Current or former		43 (15.1)
**Alcohol**		
No		276 (96.8)
Yes		9 (3.2)
**Post-surgical complications**		
None		239 (83.9)
Seromas and hematomas		46 (16.1)
**Neoadjuvant CT**		
No		278 (97.5)
Yes		7 (2.5)
**Adjuvant treatments**		
None		40 (14.2)
Chemotherapy (C)		58 (20.6)
Hormone Therapy (HT)		131 (46.5)
C+HT		53 (18.8)
**Radiation quality**		
X-rays		263 (92.3)
γ-rays		22 (7.7)
**Dose/fraction**		
2 Gy		274 (96.1)
1,8 Gy		11 (3.9)
**Electron Boost dose/fraction**		
3 Gy		72 (25.3)
1.5–2 Gy		189 (66.3)
No boost		24 (8.4)
**Acute skin toxicity, RTOG grade**		
0–1		196 (68.8)
2–3		89 (31.2)

BMI, body mass index; CI, confidence interval; CT, chemotherapy; CTV, clinical target volume; HT, hormone therapy; RTOG, Radiation Therapy Oncology Group; SD, standard deviation.

Univariate Cox regression analysis revealed significant association of age, BMI, breast diameter and vascular disease with grade ≥ 2 telangiectasia (Table 2), while BMI, breast diameter and radiation quality were found to be correlated with grade ≥ 2 subcutaneous fibrosis (Table 3). It should be noted that BMI and breast diameter were found associated to grade ≥ 2 subcutaneous fibrosis or grade ≥ 2 telangiectasia, while age and vascular disease correlated only to telangiectasia, and radiation quality only to subcutaneous fibrosis. No other clinical variable, including dose per fraction or fractionation regimen, was significantly related to grade ≥ 2 telangiectasia or grade ≥ 2 subcutaneous fibrosis.

**Table 2:** Univariate cox regression analysis for the association of clinical variables with radiation-induced grade ≥ 2 telangiectasia (SOMA-LENT scale) in breast cancer patients.

Clinical variable	Grade 0–1 n (%)	Grade ≥ 2 n (%)	HR (95% CI)	P value
**Age, years (SD)**	60.2 (10.0)	66.7 (9.0)	1.049 (1.008–1.091)	0.020
**BMI, mean (SD)**	24.8 (3.8)	26.9 (3.3)	1.095 (1.026–1.169)	0.007
**Breast diameter, cm (SD)**	12.1 (2.6)	13.2 (3.1)	1.190 (1.060–1.337)	0.003
**Breast CTV, cc (SD)**	382.7 (539.8)	536.1 (372.4)	1.000 (1.000–1.001)	0.307
**Diabetes mellitus**				
No	243 (93.8)	24 (92.3)	1 (reference)	
Yes	16 (6.2)	2 (7.7)	1.108 (0.261–4.703)	0.890
**Hypertension**				
No	188 (72.6)	23 (88.5)	1 (reference)	
Yes	71 (27.4)	3 (11.5)	0.400 (0.121–1.328)	0.137
**Vascular disease**				
No	243 (93.8)	20 (76.9)	1 (reference)	
Yes	16 (6.2)	6 (23.1)	3.238 (1.294–8.106)	0.012
**Tabagism**				
Never	218 (84.2)	24 (92.3)	1 (reference)	
Current or former	41 (15.8)	2 (7.7)	0.550 (0.130–2.326)	0.419
**Alcohol**				
No	251 (96.9)	25 (96.2)	1 (reference)	
Yes	8 (3.1)	1 (3.8)	1.142 (0.156–8.382)	0.897
**Post-surgical complication**s				
None	215 (83.0)	24 (92.3)	1 (reference)	
Seromas and hematomas	44 (17.0)	2 (7.7)	0.327 (0.077–1.391)	0.132
**Neoadjuvant C**				
No	253 (97.7)	25 (96.2)	1 (reference)	
Yes	6 (2.3)	1 (3.8)	2.218 (0.300–16.364)	0.437
**Adjuvant treatments**				
None	34 (13.3)	6 (23.1)	1 (reference)	
Chemotherapy (C)	56 (20.7)	5 (19.2)	0.792 (0.237–2.645)	0.706
Hormone Therapy (HT)	118 (46.1)	13 (50.0)	0.920 (0.341–2.482)	0.869
C+HT	51 (19.9)	2 (7.0)	0.402 (0.080–2.021)	0.271
**Radiation quality**				
X-rays	241 (93.1)	22 (84.6)	1 (reference)	
γ-rays	18 (6.9)	4 (15.4)	0.644 (0.182–2.287)	0.499
**Dose/fractio**n				
2 Gγ	251 (96.9)	23 (88.5)	1 (reference)	
1,8 Gγ	8 (3.1)	3 (11.5)	1.145 (0.315–4.165)	0.838
**Electron Boost dose/fraction**				
3 Gy	68 (26.3)	4 (15.4)	1 (reference)	
1.5–2 Gy	168 (64.9)	21 (80.8)	0.719 (0.229–2.260)	0.574
No boost	23 (8.9)	1 (3.8)	0.345 (0.038–3.144)	0.348
**Acute skin toxicity, RTOG grade**				
0–1	180 (69.5)	16 (61.5)	1 (reference)	
≥ 2	79 (30.5)	10 (38.5)	1.885 (0.853–4.167)	0.119

BMI, body mass index; CI, confidence interval; CT, chemotherapy; CTV, clinical target volume; HR, hazard ratio; HT, hormone therapy; n, number; RTOG, Radiation Therapy Oncology Group; SD, standard deviation.

**Table 3:** Univariate cox regression analysis for the association of clinical variables with radiation-induced grade ≥ 2 fibrosis (SOMA-LENT scale) in our cohort of breast cancer patients.

Clinical variable	Grade 0–1 n (%)	Grade ≥ 2 n (%)	HR (95% CI)	P value
**Age, years (SD)**	60.4 (10.0)	62.6 (10.2)	1.008 (0.981–1.037)	0.554
**BMI, mean (SD)**	24.7 (3.8)	26.1 (3.7)	1.058 (1.001–1.118)	0.048
**Breast diameter, cm (SD)**	12.1 (2.7)	12.8 (2.4)	1.126 (1.030–1.231)	0.009
**Breast CTV, cc (SD)**	383.3 (576.8)	416.7 (208.2)	1.0001 (0.9997–1.0005)	0.725
**Diabetes mellitus**				
No	221 (94.4%)	46 (90.2%)	1 (reference)	
Yes	13 (5.6%)	5 (9.8%)	1.585 (0.631–3.979)	0.329
**Hypertension**				
No	171 (73.1%)	40 (78.4%)	1 (reference)	
Yes	63 (26.9%)	11 (21.6%)	0.850 (0.437–1.652)	0.633
**Vascular disease**				
No	215 (91.9%)	48 (94.1%)	1 (reference)	
Yes	19 (8.1%)	3 (5.9%)	0.683 (0.214–2.180)	0.522
**Tabagism**				
Never	197 (84.2%)	45 (88.2%)	1 (reference)	
Current or former	37 (15.8%)	6 (11.8%)	0.820 (0.351–1.919)	0.650
**Alcohol**				
No	227 (97.0%)	49 (96.1%)	1 (reference)	
Yes	7 (3.0%)	2 (3.9%)	1.063 (0.260–4.345)	0.933
**Postsurgical complications**				
None	193 (82.5%)	46 (90.2%)	1 (reference)	
Seromas and hematomas	41 (17.5%)	5 (9.8%)	0.487 (0.194–1.222)	0.127
**Neoadjuvant CT**				
No	228 (97.4%)	50 (98.0%)	1 (reference)	
Yes	6 (2.6%)	1 (2.0%)	0.792 (0.111–5.679)	0.818
**Adjuvant treatments**				
None	34 (14.5%)	6 (11.8%)	1 (reference)	
Chemotherapy (C)	51 (21.8%)	10 (19.6%)	1.444 (0.525–3.970)	0.479
Hormone Therapy (HT)	107 (45.7%)	24 (47.1%)	1.513 (0.620–3.695)	0.365
C+HT	42 (17.9%)	11 (21.6%)	2.063 (0.759–5.609)	0.158
**Radiation quality**				
X-rays	214 (91.5%)	49 (96.1%)	1 (reference)	
γ-rays	20 (8.5%)	2 (3.9%)	0.211 (0.050–0.897)	0.036
**Dose/fraction**				
2 Gγ	224 (95.7%)	50 (98.0%)	1 (reference)	
1,8 Gγ	10 (4.3%)	1 (2.0%)	0.236 (0.032–1.725)	0.157
**Electron Boost dose/fraction**				
3 Gy	62 (26.5%)	10 (19.6%)	1 (reference)	
1.5–2 Gy	152 (65.0%)	37 (72.5%)	0.643 (0.305–1.358)	0.250
No boost	20 (8.5%)	4 (7.8%)	0.678 (0.210–2.190)	0.518
**Acute skin toxicity, RTOG grade**				
0–1	164 (70.1%)	32 (62.7%)	1 (reference)	
≥ 2	70 (29.9%)	19 (37.3%)	1.670 (0.944–2.953)	0.080

BMI, body mass index; CI, confidence interval; CT, chemotherapy; CTV, clinical target volume; HR, hazard ratio; HT, hormone therapy; n, number; RTOG, Radiation Therapy Oncology Group; SD, standard deviation.

**Fig 1:**
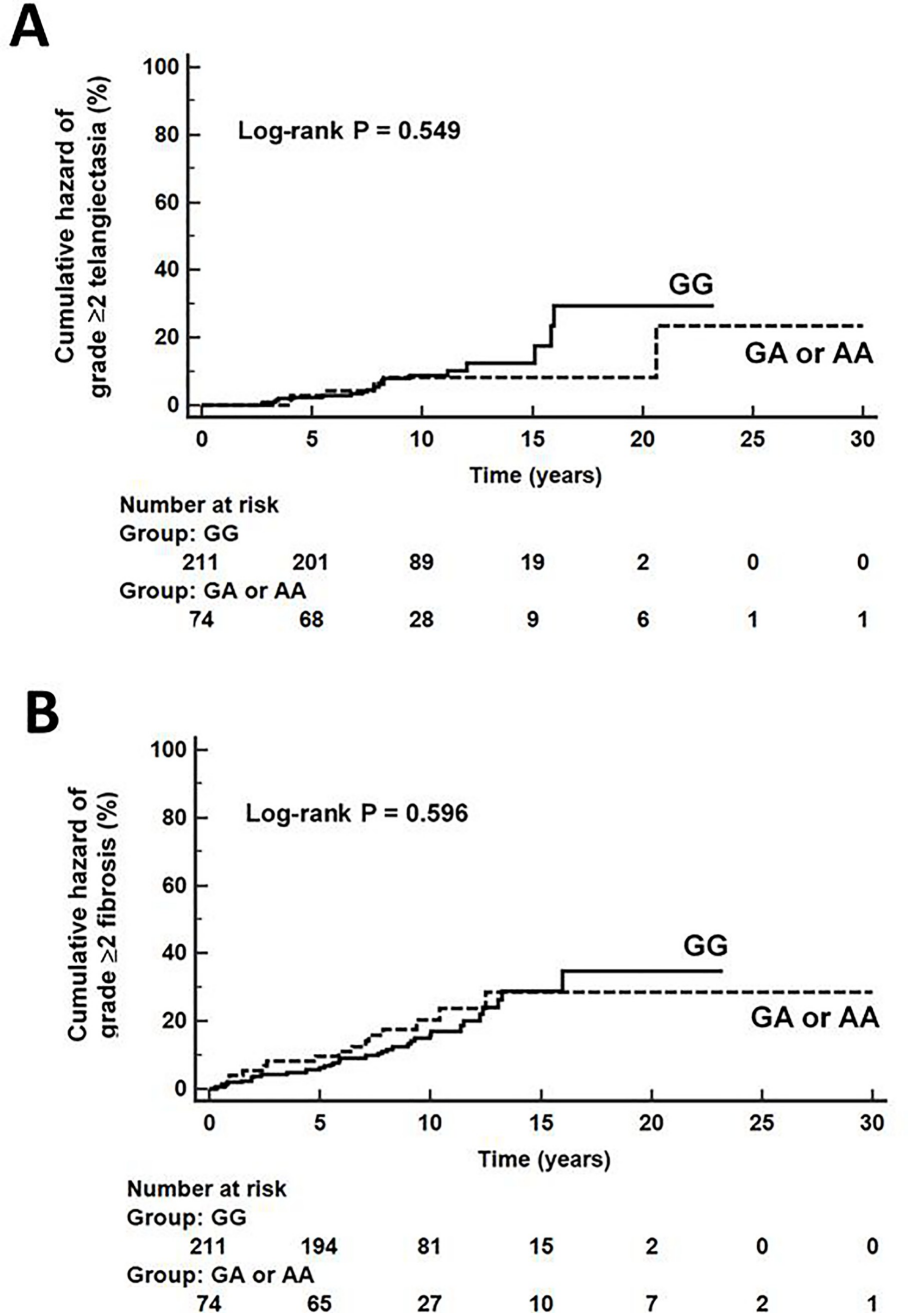
ATM rs1801516 genotypes and development of grade ≥2 radiation-induced telangiectasia (A) or grade ≥ 2 radiation-induced fibrosis (B) in our cohort of breast cancer patients.

In the entire set of breast cancer patients, the genotype frequency distribution of ATM rs1801516 was in HWE (P = 0.50) and the minor A allele frequency was of 13.7%. The Kaplan-Meier curves showed no differences among carriers and not carriers of the rs1801516 A allele in the cumulative incidence of grade ≥ 2 telangiectasia (log-rank test p-value = 0.549, Fig 1A) or grade ≥ 2 subcutaneous fibrosis (log-rank test p-value = 0.596, Fig 1B).

Cox regression analyses adjusted for confounding clinical variables revealed no association of ATM rs1801516 with grade ≥ 2 telangiectasia (GA or AA vs GG, HR_adjusted_: 0.699; 95%CI: 0.273–1.792, P = 0.459, Table 4) or with grade ≥ 2 fibrosis (GA or AA vs GG, HR_adjusted_: 1.175; 95%CI: 0.641–2.154, P = 0.604, Table 4).

**Table 4:** Cox regression analysis for the association of ATM rs1801516 with radiation-induced late skin injuries (LENT-SOMA scales) in our cohort of breast cancer patients.

**Genotype**	**Telangectasia**		**Unadjusted analysis**		Adjusted analysis^*^	
	**Grade 0–1, n (%)**	**Grade ≥2, n (%)**	**HR (95% CI)**	**P value**	**HR (95% CI)**	**P value**
GG	191 (73.7)	20 (76.9)	1 (reference)		1 (reference)	
GA	65 (25.1)	5 (9.2)	0.678 (0.252–1.823)	0.444	0.612 (0.224–1.672)	0.341
AA	3 (1.2)	1 (3.8)	2.025 (0.250–16.401)	0.511	2.716 (0.344–21.444)	0.346
GA+AA	68 (26.3)	6 (23.1)	0.751 (0.296–1.908)	0.550	0.699 (0.273–1.792)	0.459
**Genotype**	**Fibrosis**		**Unadjusted analysis**		**Adjusted analysis**^**#**^	
	**Grade 0–1, n (%)**	**Grade ≥2, n (%)**	**HR (95% CI)**	**P value**	**HR (95% CI)**	**P value**
GG	175 (74.9)	36 (70.6)	1 (reference)		1 (reference)	
GA	56 (23.9)	14 (27.5)	1.158 (0.626–2.141)	0.642	1.144 (0.616–2.124)	0.672
AA	3 (1.3)	1 (2.0)	1.537 (0.211–11.167)	0.673	2.064 (0.270–15.754)	0.487
GA or AA	59 (25.2)	15 (29.4)	1.177 (0.646–2.145)	0.597	1.175 (0.641–2.154)	0.604

*Adjusted by age, BMI, breast diameter and vascular disease

^#^adjusted by BMI, breast diameter and radiation quality. Abbreviations: HR, hazard ratio; 95% CI, 95% confidence interval.

### Systematic review and meta-analysis

**Fig 2:**
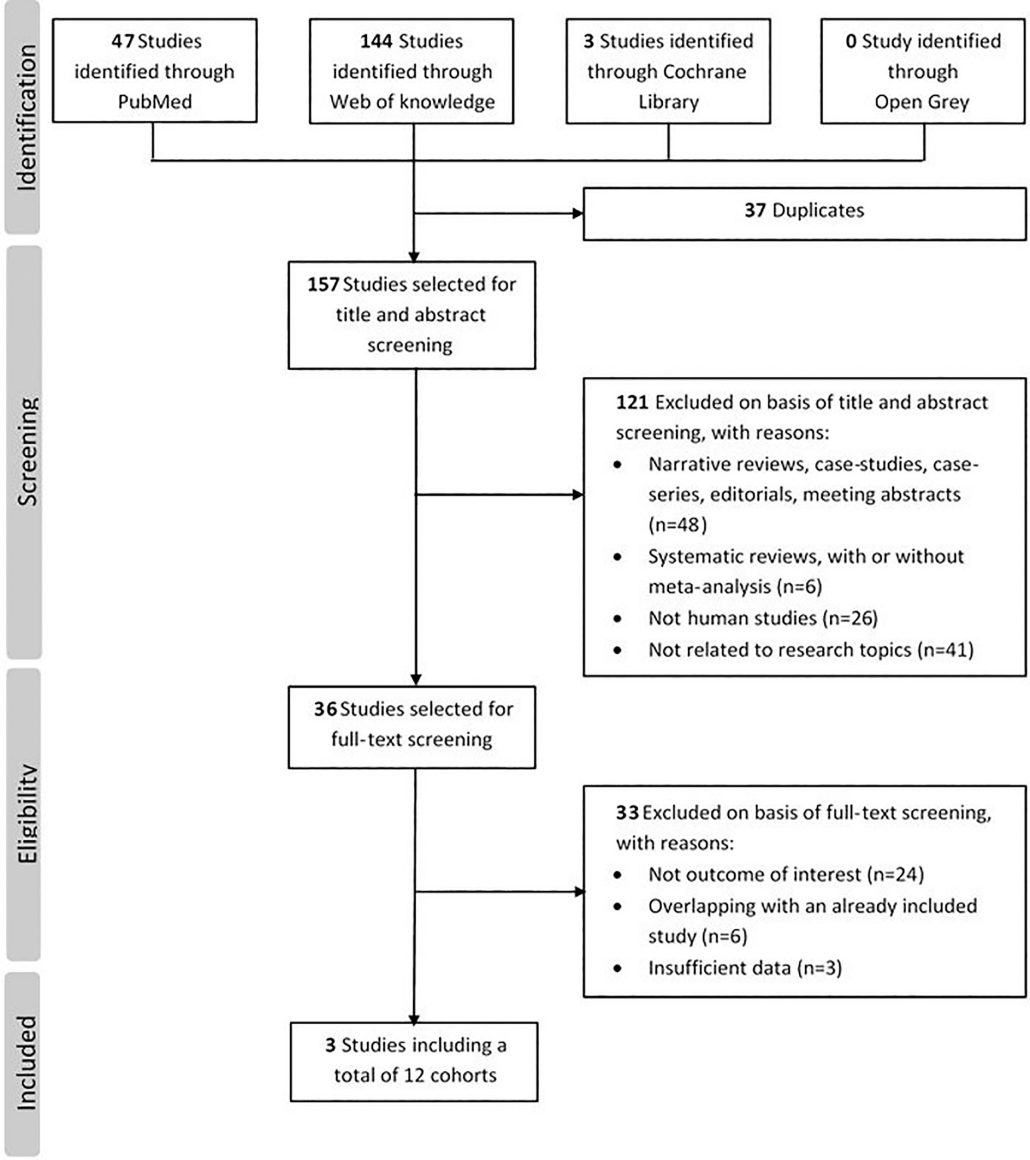
Flowchart of literature search and study selection.

The electronic search in PubMed, Web of Knowledge, Cochrane and Open Grey yielded a total of 194 records, of which 37 were duplicates. After removal of additional 154 hits not fulfilling inclusion criteria, 3 studies including a total of 12 independent cohorts of breast cancer patients were identified through the systematic review [32–34]. The flowchart of the literature review process is reported in Fig 2. The identified studies were published from 2007 to 2016, and the sample sizes ranged from 41 to 940.

**Fig 3:**
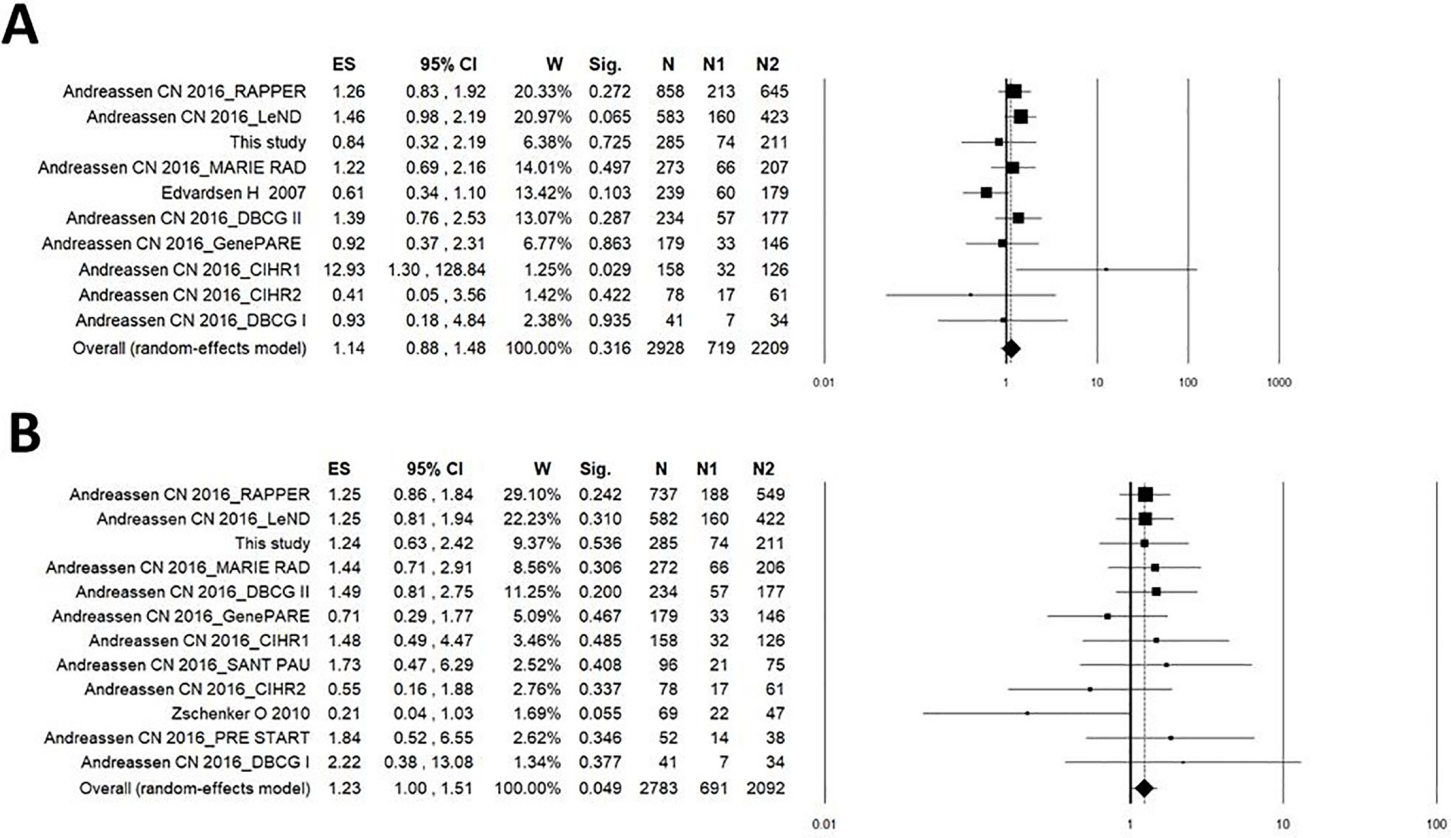
**Forest plot for the association between ATM rs1801516 (GA or AA vs GG) and radiation-induced telangiectasia (A) or radiation-induced fibrosis (B) in breast cancer patients.** ES, effect size (i.e. odd ratio); W, weight; Sig, statistical significance; N, total number of breast cancer patients; N1, number of carriers of the minor allele of rs1801516 (GA or AA); N2, number of GG genotype carriers.

**Fig 4:**
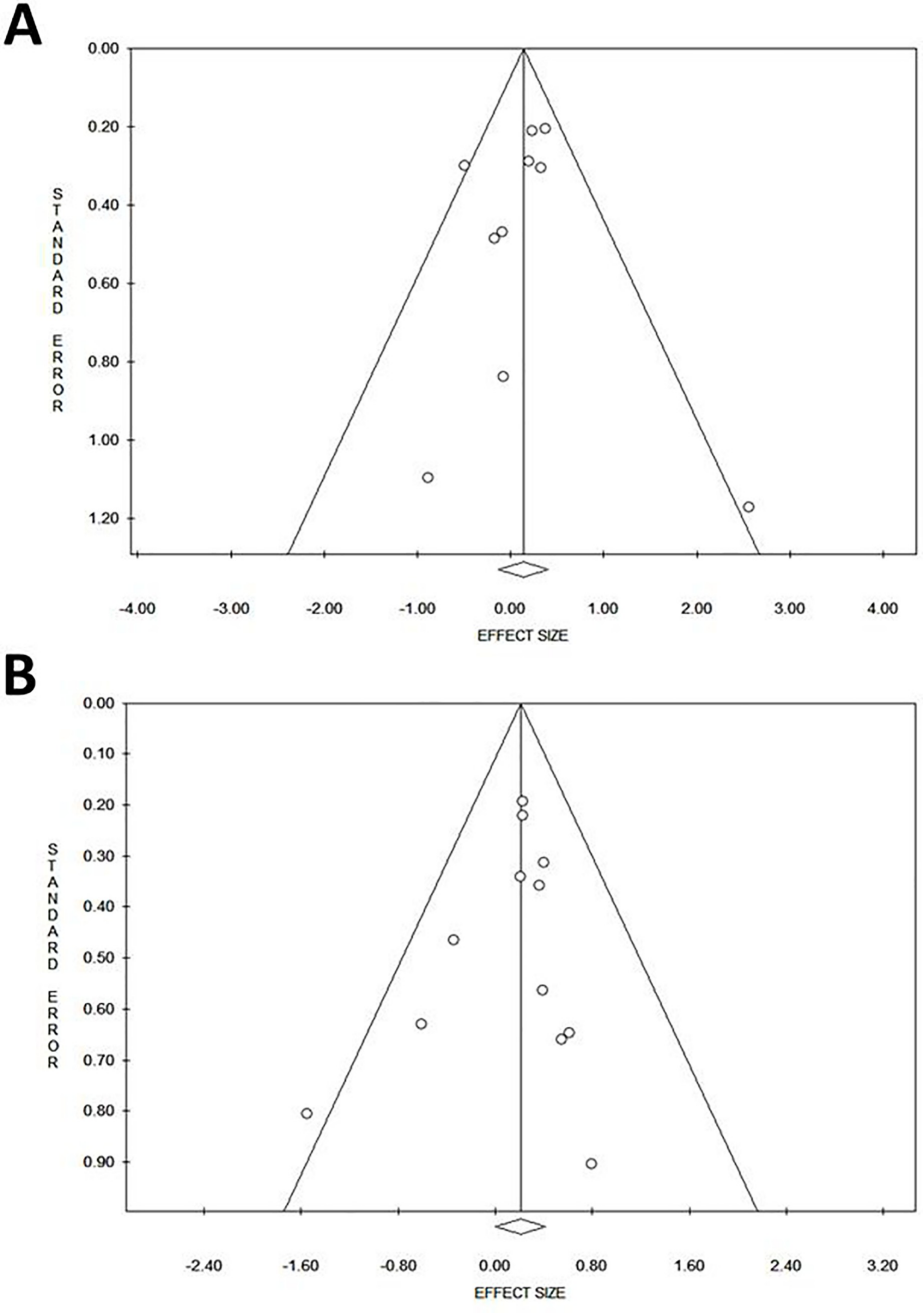
Funnel plot for the effect of ATM rs1801516 on radiation-induced telangiectasia (A) or fibrosis (B) in breast cancer patients.

Detailed characteristics of previously published studies, reporting data in breast cancer patients on the association between ATM rs1801516 and radiation-induced telangiectasia or fibrosis, are presented in Table 5. The study of Edvardsen et al. [32], with a NOS score of 5, was considered of lower methodological quality compared to the other studies included in the systematic review, having a NOS score of 8 (Table 5). Overall, 10 cohorts of breast cancer patients (total n = 2928) were included in the meta-analysis of ATM rs1801516 and risk of developing radiation-induced telangiectasia. The pooled analysis showed no significant heterogeneity between studies (I^2^ = 25.8%; P = 0.206) and a non-significant effect of ATM rs1801516 on the risk of breast cancer patients to develop radiation-induced telangiectasia (pooled OR: 1.14; 95%CI: 0.88–1.48, P = 0.316; Fig 3A). In addition, no evidence of publication bias or small-study effects was observed both in the funnel plot (Fig 4A) and in the Egger’s test (P = 0.824).

**Table 5:** Characteristics of studies included in the systematic review.

First Author, year [Ref]	Cohort name	Country	Sample size	Dose, Gy	Scoring system	Cut-off for case definition	Late skin injury (cases/total)	Follow-up, y (range)	Detection method	MAF/ HWE p-value	NOS score
Edvardsen H, 2007 [32]	-	Norway	367	43 or 50	CTCAE or LENT-SOMA or ad hoc graded scale	≥ Grade 2	RIT (141/239)	NR	dHPLC + Seq	0.14/0.75	5
Zschenker O, 2010 [33]	-	Germany	69	54–55	LENT-SOMA	≥ Grade 2	RIF (17/69)	12 (9–20)	MassArray	0.17/0.94	8
Andreassen CN, 2016 [34]	CIHR1	Canada	158	42.40 42.56 or 50	CTCAE (v3.0)	Upper quartile score	RIF (19/158)	2	MassArray + Seq	0.12/0.03	8
							RIT (4/158)				
	CIHR2	Canada	78	42.40 42.56 or 50	CTCAE (v3.0)	Upper quartile score	RIF (26/78)	2	MassArray + Seq	0.12/0.97	
							RIT (9/78)				
	DBCG I	Denmark	41	36.6–51.4	LENT-SOMA	Upper quartile score	RIF (23/41)	2.2–5.4	dHPLC + Seq	0.11/0.02	
							RIT (24/41)				
	DBCG II	Denmark	234	36.6–51.4	LENT-SOMA	Upper quartile score	RIF (82/234)	2.1–5.8	TaqMan real-time PCR	0.12/0.12	
							RIT (117/234)				
	GenePARE	France, Switzerland, USA	181	45–50	CTCAE (v3.0) or RTOG/EORTC	Upper quartile score	RIF (47/179)	3–6	Affymetrix 6.0 array or dHPLC + Seq	0.10/0.81	
							RIT (40/179)				
	LeND	UK	602	50	LENT-SOMA	Upper quartile score	RIF (122/582)	5.2	SNPlex	0.15/0.54	
							RIT (147/583)				
	MARIE_RAD_	Germany	273	50–56	RTOG/EORTC	Upper quartile score	RIF (210/272)	3.7–7.5	Illumina GoldenGate Assay	0.13/0.79	
							RIT (98/273)				
	Pre START	UK	52	50 or 42.9 or 39.0	Ad hoc graded scale	Upper quartile score	RIF (17/52)	5	PCR and SNaPshot primer extension	0.16/0.10	
	RAPPER*	UK	940	40	LENT-SOMA	Upper quartile score	RIF (173/737)	2	CytoSNP 12 microarray	0.14/0.75	
							RIT (129/858)				
	SANT PAU	Spain	101	50	RTOG	Upper quartile score	RIF (13/96)	2	Sanger Seq	0.12/0.18	
This study	-	Italy	285	50	LENT-SOMA	≥ Grade 2	RIF (51/285)	12 (4–31)	TaqMan real-time PCR	0.14/0.50	8
							RIT (26/285)				

*ATM rs1801516 was indirectly assessed by means of a proxy SNP (rs4988023, r^2^ = 1). Abbreviations: CTCAE: Common Toxicity Criteria for Adverse Events; dHPLC: denaturing High Performance Liquid Chromatography; LENT-SOMA: Late Effects of Normal Tissues–Subjective Objective Management Analytic; MAF: minor allele frequency of rs1801516; NOS: Newcastle–Ottawa scale; NR: not reported; RIF: radiation-induced fibrosis; RIT: radiation-induced telangiectasia; RTOG/EORTC: Radiation Therapy Oncology Group/European Organization for Research and Treatment of Cancer morbidity criteria; Seq: sequencing.

**Fig 5:**
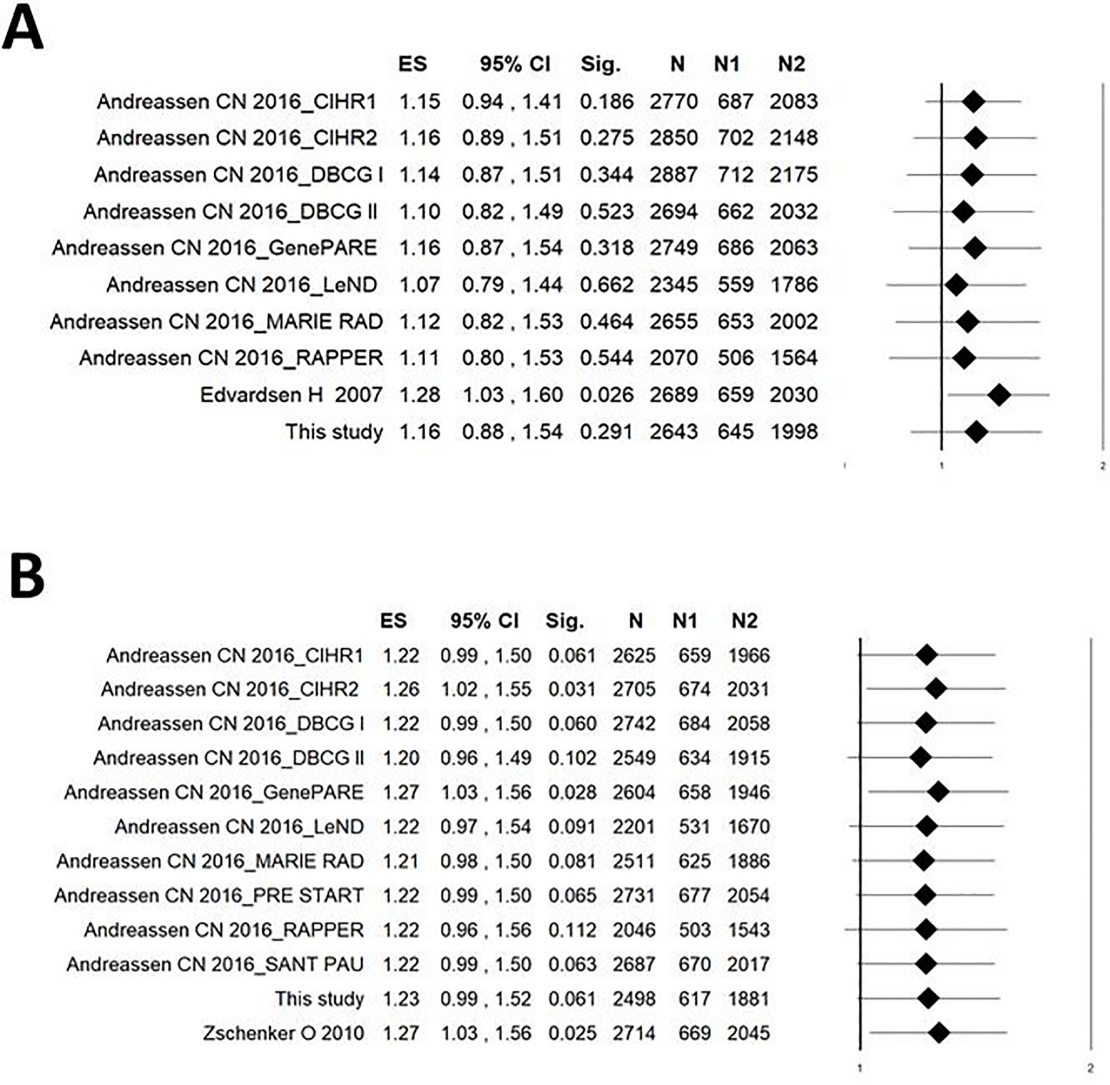
**Leave-one-out sensitivity analysis for the effect of ATM rs1801516 (GA or AA vs GG) on radiation-induced telangiectasia (A) or radiation-induced fibrosis (B), by excluding individual studies one at a time and recalculating the pooled odd ratio estimate for the remaining studies.** ES, effect size (i.e. odds ratio); Sig, statistical significance; N, total number of breast cancer patients; N1, number of carriers of the minor allele of rs1801516 (GA or AA); N2, number of GG genotype carriers.

However, leave-one-out sensitivity meta-analysis (Fig 5A) showed a significant association of ATM rs1801516 (OR: 1.28; 95% CI: 1.03–1.60, P = 0.026) when 1 study with NOS of 5 was excluded due to lower methodological quality score [32], suggesting lack of robustness of the overall pooled estimate. Meta-regression analysis showed lack of a linear relationship between OR and incidence of telangiectasia (P = 0.285).

A total of 12 cohorts (total n = 2783) were available for the meta-analysis of ATM rs1801516 and risk of developing radiation-induced fibrosis. Results of this pooled analysis showed lack of heterogeneity between studies (I^2^ = 0%; P = 0.570) and a borderline significant effect of ATM rs1801516 (pooled OR: 1.23; 95%CI: 1.00–1.51, P = 0.049; Fig 3B). The funnel plot (Fig 4B) and the Egger’s test (P = 0.423) provided no evidence of publication bias or small-study effects. Results of leave-one-out sensitivity meta-analysis showed instability of the pooled OR estimate, with effect sizes ranging from 1.20 (95% CI: 0.97–1.49, P = 0.101) to 1.27 (95% CI: 1.03–1.56, P = 0.025) (Fig 5B). Meta-regression analysis showed no relationship between OR and incidence of fibrosis (P = 0.622).

### Trial sequential analysis (TSA)

**Fig 6:**
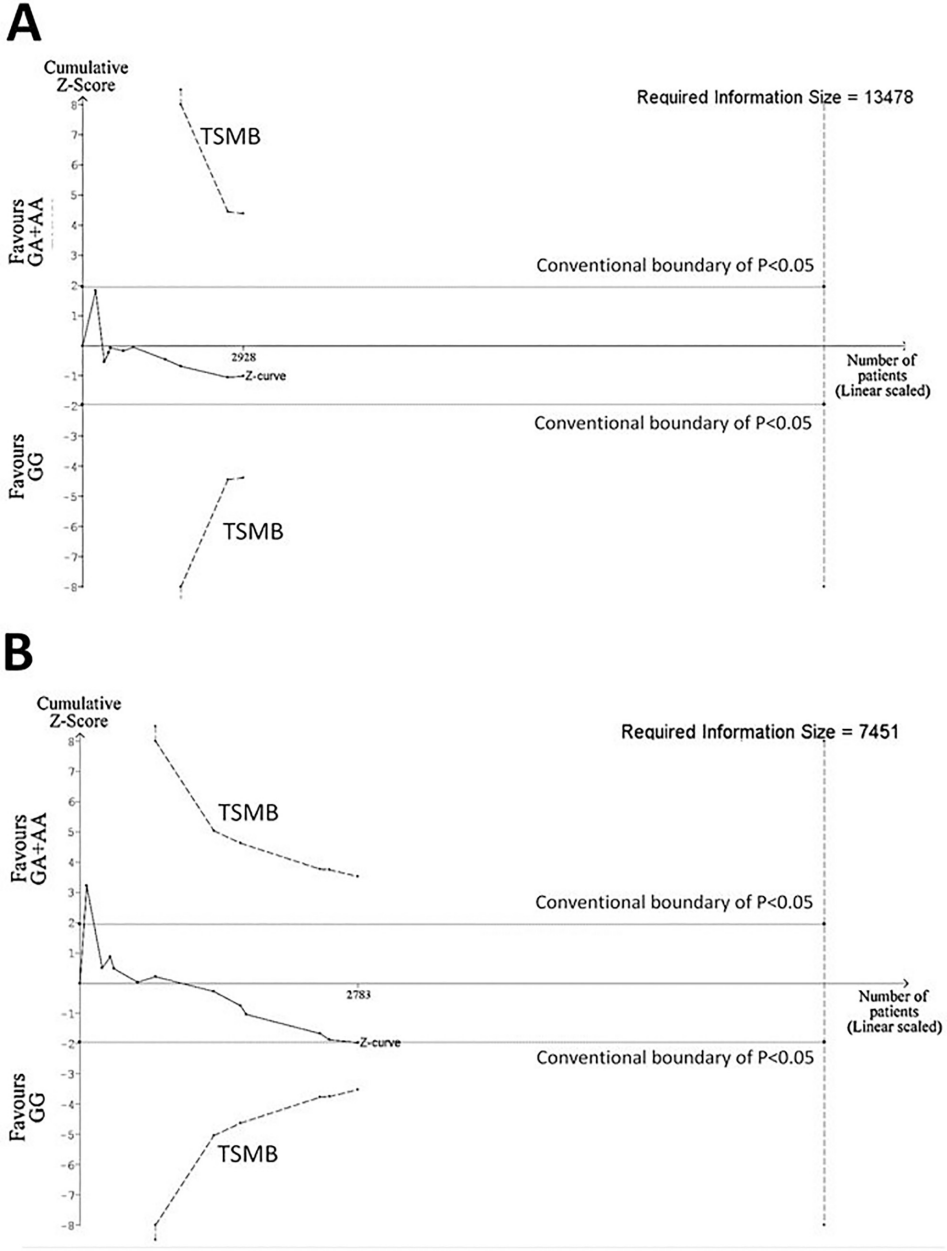
**Trial sequential analysis for ATM rs1801516 (GA or AA vs GG) and risk of developing radiation-induced telangiectasia (A) or radiation-induced fibrosis (B), in breast cancer patients.** In both analyses, the Z-curve does not cross the trial sequential monitoring boundary (TSMB) and the required information size (i.e. sample size) was not achieved, suggesting that there is still insufficient evidence to draw definite conclusions on the effect of ATM rs1801516.

TSA for radiation-induced telangiectasia showed that the cumulative z-curve did not cross the trial sequential monitoring boundary (TSMB) nor did it reach the required information size (RIS = 13478 participants, Fig 6A). Similarly, TSA for radiation-induced fibrosis revealed that the cumulative z-curve did not cross the TSMB, the required information size was not achieved (RIS = 7451 participants, Fig 6B) and that finding of conventional meta-analysis was a false positive result (TSA-adjusted 95%CI: 0.85–1.78). Overall, these findings suggest that there is still insufficient evidence to draw definitive conclusions on the correlation between ATM rs1801516 and late radiation skin injuries in breast cancer patients.

## Discussion

Over the last decade, a number of candidate gene association studies has been conducted in cancer patients to assess the role of the ATM rs1801516 gene polymorphism as risk factor for normal tissue complications of radiotherapy, in particular in prostate cancer, however inconsistent results have been reported due to insufficient statistical power of most studies [33, 35–37]. Looking at another pathology with high incidence and health impact as breast cancer, we attempted to evaluate the association of rs1801516 with late skin side effects of radiotherapy for breast cancer. Late skin radiation-induced reactions should be considered separately from acute reactions because they affect different tissue components (derma vs. epidermis) with different pathogenesis (mainly through microvascular damage vs. direct cell damage) and can result permanent whereas acute are usually temporary. In addition, fibrosis and telangiectasia may be severe with an impact on breast cosmetic outcome and on patient physical well-being and quality of life [4–5]. In the present study we assessed the role of ATM rs1801516 as predictive factor for fibrosis and telangiectasia in a cohort of breast cancer patients who received radiotherapy after breast conserving surgery. We then conducted a systematic review and a trial meta-analysis to establish whether the accumulated evidence on the relationship between rs1801516 and radiation-induced late skin side effects of radiotherapy for breast cancer might be sufficient for firm conclusions.

In our cohort of 285 breast cancer patients we found no association between ATM rs1801516 and radiation-induced fibrosis or telangiectasia. These results are in line with those of most of the cohorts included in the present systematic review, and consistent with the pooled results on risk of radiation-induced telangiectasia. With regard to predictive clinical variables, it can be argued that telangiectasia can be correlated to the skin dose and in particular to the dose to most superficial layers of the skin. However, in the present patient series, skin was relatively preserved at the level of the boost volume since patients received 9 MeV electrons without bolus, meaning that surface skin dose (about 85% of the maximum dose) was not able to significantly influence the development of telangiectasia. As a matter of fact, age, breast diameter, BMI and vascular disease but not the electron boost were correlated with the onset of telangiectasia. As regard to radiation fibrosis, the meta-analysis including 2783 breast cancer patients revealed a borderline significant association of ATM rs1801516, being the risk higher among carriers of the minor A allele (OR: 1.23; 95%CI: 1.00–1.51, P = 0.049). This result is in line with a previous meta-analysis including only 1932 breast cancer patients [21], which reported for carriers of the rs1801516 A allele an OR of 1.80 (95%CI:1.03–3.14, P = 0.040), and also consistent with results of the International Radiogenomics Consortium [34], which reported an OR of 1.27 (95%CI: 1.02–1.58) in the pooled analysis of 2429 breast cancer patients. Nevertheless, our TSA results clearly showed that the postulated association of rs1801516 with radiation-induced fibrosis in breast cancer may be a false positive result and that the current number of breast cancer patients in the pooled analysis is considerably lower than that required for a sufficiently well powered study.

ATM rs1801516 is known to result in the substitution of aspartic acid to asparagine at position 1853 (D1853N), thus it may have a functional effect on the gene product. Nevertheless, predictive algorithms classify rs1801516 as a “benign” or “likely-benign” polymorphism, as reported by NCBI ClinVaR and SNPeffect databases (available at https://www.ncbi.nlm.nih.gov/clinvar and http://snpeffect.switchlab.org/sequences, respectively). In addition, no significant differences were observed in constitutive ATM protein levels, cell survival or p53 protein induction, after IR exposure of lymphoblastoid cell lines from breast cancer patients carrying 0, 1 or 2 minor alleles of rs1801516 [18]. On the other hand, no association of ATM rs1801516 with breast cancer susceptibility was reported by genome-wide association studies repertoried in the GWAS Catalog website (available at https://www.ebi.ac.uk/gwas), while a recent meta-analysis of case-control candidate gene association studies found a decreased risk of breast cancer among subjects carrying the rs1801516 AA genotype [38]. Based on these and our findings, it is safe to conclude that the clinical relevance of ATM rs1801516 remains uncertain, but it deserves further investigation. However, genotyping of ATM rs1801516 SNP alone may not necessarily represent a good strategy for prediction of normal tissue radiosensitivity and other approaches based on immunofluorescence or ELISA detection of the ATM protein may be more clinically useful. The ATM kinase enzyme is mainly located in the cytoplasm as dimers formed by two autophosphorylated (pATM) monomers at serine 1981, and ionizing radiation induces the monomerization of cytoplasmic ATM dimers and triggers their diffusion in the nucleus to recognize and repair DNA double-strand breaks [39]. A pATM ELISA assay, based on the quantification of the nuclear forms of autophosphorylated ATM protein, has been recently reported to discriminate radioresistant and radiosensitive patients with very high statistical performances [40]. This assay, which was conducted on 30 skin fibroblasts from 9 radioresistant (toxicity grade<2) and 21 radiosensitive (toxicity grade ≥2) patients, showed an average AUC value higher than 0.8, a sensitivity of 0.8 and a specificity ranging from 0.75 and 1 [40]. While these results document the potential predictive power of the pATM ELISA assay, these discrimination performances must be confirmed in a larger number of patients for prediction of both early and late skin radiation-induced effects. On the other hand, clinical radiosensitivity is currently regarded as a complex trait resulting from the combined effects of multiple genetic factors, each with relatively modest effects [6]. Given that the predictive power provided by a single polymorphism has been demonstrated to be rather modest [41–43], an approach based on the combination of multiple loci into a global genetic risk score (GRS) may be a more attractive strategy than the single SNP approach for prediction of adverse radiotherapy effects. Therefore, future studies should also evaluate clinical utility of ATM rs1801516 incorporation in GRS-based models for prediction of late skin radiation-induced effects.

A few limitations and considerations should be acknowledged in the present study. First, we performed a retrospective cohort study with limited sample size, nevertheless it represents the third larger cohort of breast cancer patients included in the pooled analyses. Secondly, the cohorts comprised in the present systematic review displayed marked clinical variability in terms of radiotherapy dose, scoring system of late skin side effects and relative cut-off values used for cases definition. Nevertheless, there was no statistical heterogeneity in the meta-analyses, which suggests a quite uniform effect of ATM rs1801516, regardless of clinical variability including scoring system and follow-up. Furthermore, in spite of primary studies showing a varied incidence of late skin events, results of meta-regression analyses excluded an impact of event incidence on the pooled estimates. Lastly, we acknowledge that our pooled estimates were based on few additional studies in comparison to the largest meta-analysis so far conducted [34]. However, the present updated meta-analysis with trial-sequential analysis shows for the first time that there is still insufficient information to draw reliable conclusions regarding the correlation between ATM rs1801516 and late skin toxicities induced by radiotherapy in breast cancer patients.

## Conclusions

Our cohort study does not support an impact of ATM rs1801516 on radiation-induced late skin injuries in breast cancer patients. Nevertheless, results of our meta-analysis with TSA suggest that further large studies are needed to get more reliable conclusions. These findings should therefore encourage further research on the role of ATM rs1801516 on normal tissue radiosensitivity of breast cancer patients. Nevertheless, even if conclusive evidences will be provided, the effect size of ATM rs1801516 is expected to be not large enough to be clinically useful, at least when used as single genetic marker. In the next few years, GWAS datasets derived from increasingly larger international collaborative networks should hopefully identify relevant SNPs for the construction of GRS-based models, which may be clinically useful for identification of breast cancer patients at higher risk of late skin radiation-induced effects.

## Supporting information

S1 Checklist (DOC)
